# Supplementary figures and images for: Local adaptation drives the diversification of effectors in the fungal wheat pathogen Parastagonospora nodorum in the United States
Source: PLoS Genet. 2019 Oct 18;15(10):e1008223. doi: 10.1371/journal.pgen.1008223 (PMC6821140; doi:10.1371/journal.pgen.1008223)

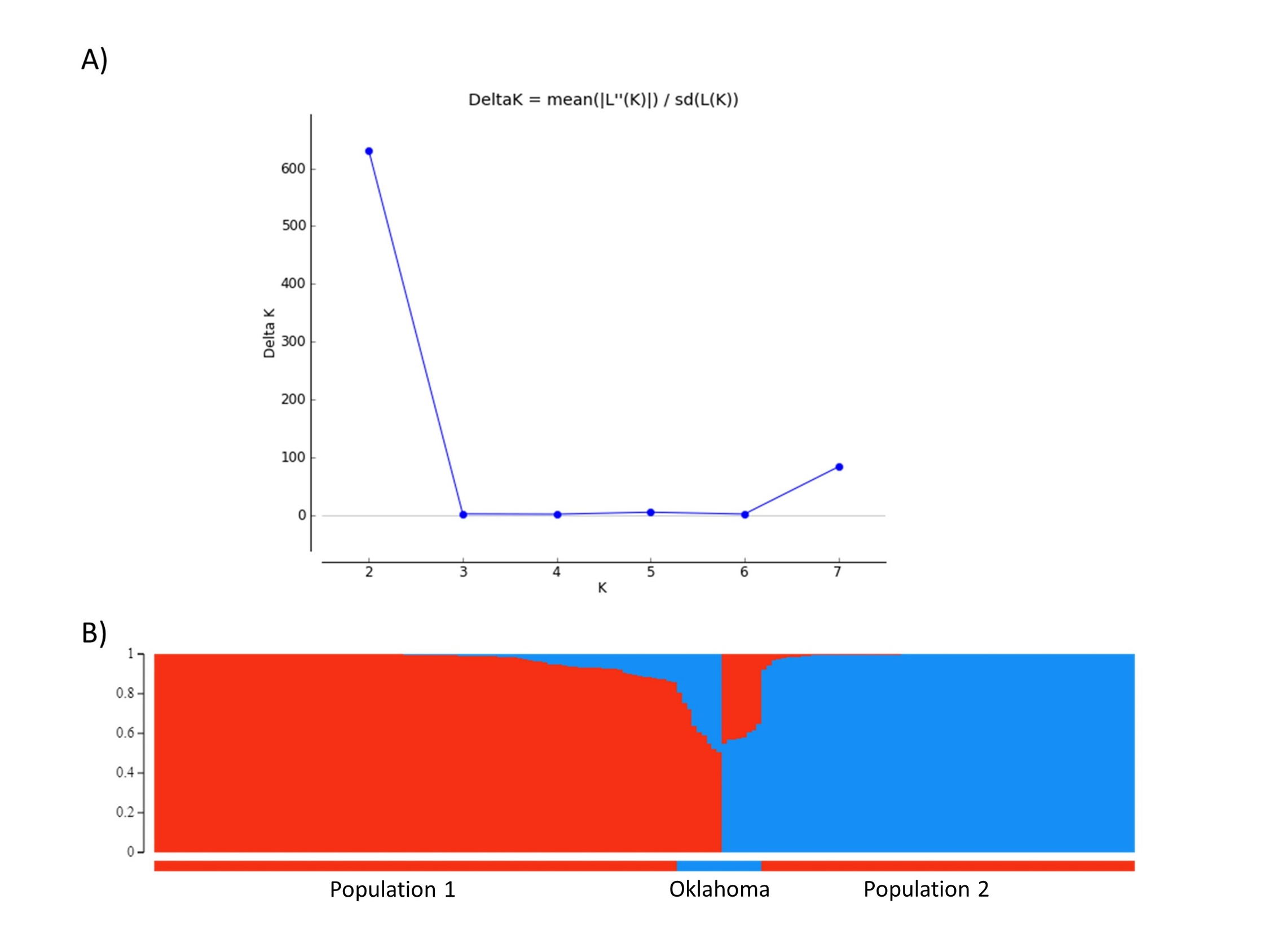

Supplement: S1 Fig — A) Evanno method indicated the optimal k value (number of subpopulations) to be two. B) Using a k-value of two, the 197 P. nodorum natural population is divided into two populations. Population 1 (red) consists of isolates from the Midwestern United States. Population 2 (blue) consists of isolates from the Southern/Eastern region of the United States, as well as Oregon. (TIF) [file pgen.1008223.s001.tif]

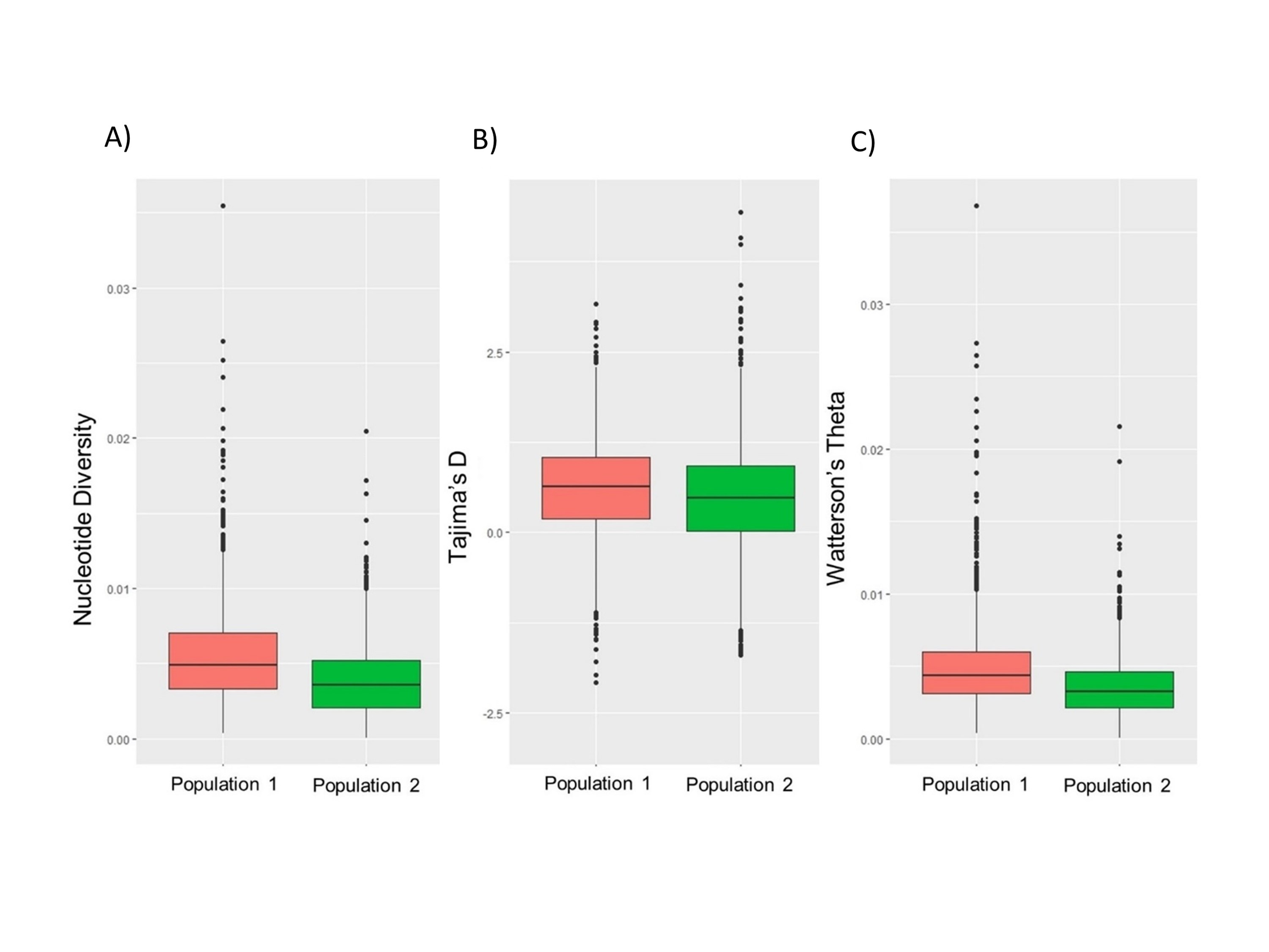

Supplement: S2 Fig — (TIF) [file pgen.1008223.s002.tif]

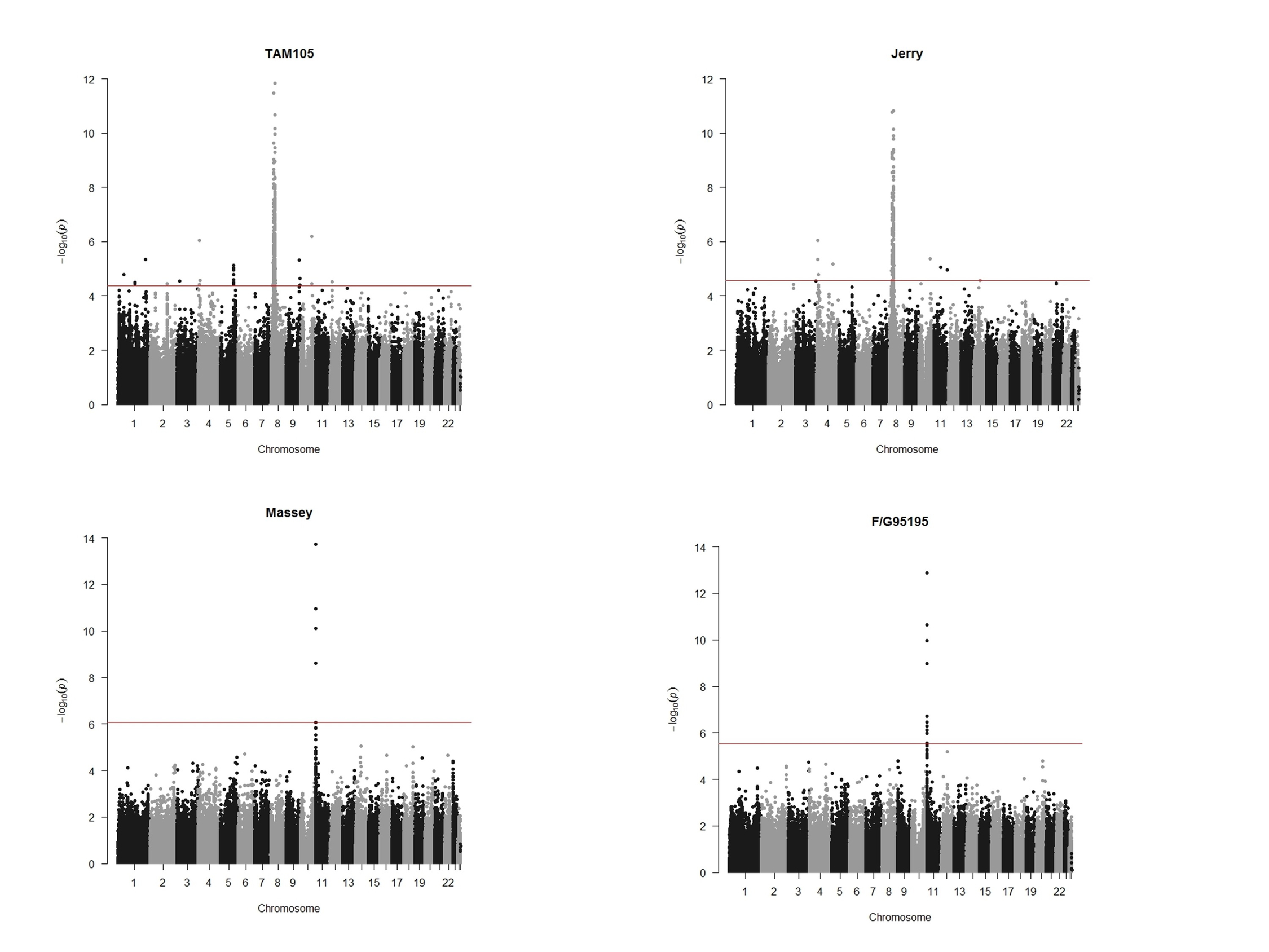

Supplement: S3 Fig — Dots represent individual SNPs/InDels. Markers are ordered by position and chromosomes are displayed on the x-axis. The–log10(p) value is displayed on the y-axis. The horizontal line represents the significance threshold at and FDR adjusted p-value of 0.05. (TIF) [file pgen.1008223.s003.tif]
